# Effectiveness of a Customized Peritoneal Dialysis Training Program in Reducing Infection and Dropout Rates: Insights From a Singapore Hospital

**DOI:** 10.7759/cureus.67997

**Published:** 2024-08-28

**Authors:** Pei Shan Lee, Hui Boon Tay, XiaoHong Luo, Seow Yean Chiang, Sing Ping Loh, Boon Cheok Lai

**Affiliations:** 1 Department of Nephrology, Sengkang General Hospital, Singapore, SGP; 2 Department of Nursing, Sengkang General Hospital, Singapore, SGP; 3 Department of Medical Social Service, Sengkang General Hospital, Singapore, SGP; 4 Department of Internal Medicine, Sengkang General Hospital, Singapore, SGP

**Keywords:** peritoneal dialysis drop out rate, peritoneal dialysis peritonitis, exit site infection, peritoneal dialysis training, peritoneal dialysis

## Abstract

Introduction

Peritoneal dialysis (PD) is an essential home-based treatment for end-stage kidney disease, known for enhancing patients' quality of life and being more cost-effective compared to hemodialysis. However, in Singapore, PD education lacks of standardization, with each unit adopting varied methods based on their own experiences and resources. To address this, our hospital developed a tailored four-day PD training program guided by the International Society for Peritoneal Dialysis guidelines, adapted to meet local needs and resource availability.

Methodology

This study employed a retrospective cohort design, including all incident adult patients aged 18 years and above who initiated PD at our hospital from September 2018 to July 2023. Data on PD dropout rates and PD-related infection rates, such as PD peritonitis and exit site infection rates, were obtained from electronic medical records.

Results

This study comprised 99 patients who began PD and completed their PD training program at our hospital between September 2018 and July 2023. Our tailored PD training program successfully reduced dropout rates and maintained infection rates within the International Society for Peritoneal Dialysis guidelines. Specifically, exit site infection rates fluctuated between 0.18 and 0.29 episodes per year, PD peritonitis rates ranged from 0.2 to 0.26 episodes per patient-year, and dropout rates significantly improved from 40% in 2019 to 7% in 2023 (OR = 0.45, 95% CI = 0.49 to 0.84, p = 0.010).

Conclusions

The tailored PD training program at our hospital effectively reduced PD-related infections and dropout rates among end-stage kidney disease patients. These findings suggest that structured, locally adapted training programs can substantially improve patient outcomes in PD.

## Introduction

Peritoneal dialysis (PD) is a crucial treatment modality for managing end-stage kidney disease (ESKD), providing a flexible and effective alternative to hemodialysis. As a home-based therapy, PD significantly enhances quality of life by enabling patients to administer treatment at home, reducing hospital visits. Additionally, PD is generally more cost-effective, alleviating the financial burden on patients and healthcare systems [1,2]. However, home nursing support varies by country, meaning many patients and caregivers must manage the treatment independently, underscoring the need for comprehensive patient education and support systems [3,4]. Simulation learning experiences have been shown to be effective in educating patients with chronic conditions, and providing practical and immersive training that enhances learning outcomes [5].

In Singapore, PD training and education for patients and caregivers lacks standardization, with each unit developing methods based on experience and resources. We are a regional hospital, which is a 1000-bed acute care hospital, that began operations in August 2018. This study shares our experience establishing a comprehensive training program for PD patients at our hospital, evaluating its effectiveness by examining PD-related infection rates, including exit site infections (ESI) and PD peritonitis, and dropout rates. By analyzing these parameters, we aim to provide insights into the impact of structured education on patient outcomes [1,6].

The International Society for Peritoneal Dialysis (ISPD) released a comprehensive guide in 2016 offering a syllabus for teaching PD to patients and caregivers, structured as a five-day program with a detailed checklist for assessing patient readiness [1,7]. Despite the availability of this syllabus, training practices for PD patients and caregivers vary globally and within Singapore. At our hospital, we developed a four-day PD training program totaling approximately 25 hours, aligning with ISPD recommendations but tailored to local needs. Our program includes opportunistic teaching during patients' return visits for wound care and PD catheter flushing post-operation, reducing hospital time for patients and caregivers. This approach aims to make training more accessible and less burdensome while ensuring comprehensive education [8,9].

This study describes the implementation of this tailored training program and evaluates its impact on PD-related infection rates, including exit site infections and peritonitis, as well as dropout rates. Preliminary results indicate the program's success in maintaining low infection rates and reducing dropout rates, demonstrating the effectiveness of structured, patient-centric training. By sharing our findings, we hope to contribute to the broader discourse on PD management and offer insights that other institutions can adopt to enhance their PD training programs and patient outcomes.

## Materials and methods

Participants and study design 

This is a retrospective cohort study conducted at a regional hospital in Singapore from September 2018 to July 2023. This retrospective cohort study was reported based on the Strengthening the Reporting of Observational Studies in Epidemiology (STROBE) checklist for cohort studies. Patients included in the study were incident adult end-stage kidney disease patients aged 18 years, who had their PD catheter inserted and started PD at our hospital, completed the entire PD training course, and continued dialysis for at least three months. Exclusion criteria included patients who did not complete the training program or discontinued dialysis before three months.

PD training program in our hospital

Motivation for Introducing a Modified PD Training Syllabus

When our hospital’s PD program started in September 2018, the team developed a four-day formal PD training program based on ISPD’s guidelines. Anticipating that most learners would be full- or part-time working individuals, the team aimed to create a more succinct program to minimize the time spent in the hospital. This formal training was supplemented with opportunistic teaching of theoretical knowledge during patients' return visits for blood work and PD catheter flushing. Additionally, written information was provided for patients and caregivers to read before the formal program commenced. Although the total hours dedicated to PD training exceed those in ISPD’s program, this approach reduces the number of hospital visits and commuting times for patients and caregivers.

Learning Objectives, Plans, and Evaluation

Training is conducted by a PD nurse, with each course taught one-on-one by the same trainer to ensure consistency and undivided attention to the learner. Throughout the process, the objectives and progress are shared with the learner. The trainer demonstrates and supervises all procedural practices to provide immediate feedback. A training log tracks the learner’s progress each day, aligned with the ISPD PD Training 2016 recommendations [1]. The main objectives are that the patient and/or caregiver can safely perform PD procedures using an aseptic technique, recognize contamination and take appropriate follow-up action, identify changes in fluid balance and its relationship to hypertension/hypotension, and detect, report, and manage potential dialysis complications using available resources, including contacting the PD unit for assistance or presenting to the emergency department appropriately for medical attention.

Course Description

Our training program begins the day after PD catheter insertion and continues during the patient's subsequent visits to the hospital for catheter flushing and dressing changes, differing from the ISPD-recommended approach.

Appendix 1 in the Appendices provides a summary table of the teaching syllabus, organized by day. Each day usually starts with a debriefing session and an informal assessment of the learner's skills and knowledge retention, enabling the course to be customized to fit the abilities and needs of each learner.

Training takes place in the PD Training Center (PDTC), an outpatient facility. In some cases, particularly for patients who have undergone urgent peritoneal dialysis catheter insertion, training is conducted on an inpatient basis while they receive treatment. Trainers adapt their methods to meet the needs of both patients and caregivers, using a variety of learning tools such as printed manuals and mannequins to enhance the training experience.

Expectations for Learners

Learners are expected to attend each training session as scheduled and primed that there may be a need to extend the training duration, based on the trainer’s assessment of their abilities. A home visit is scheduled four days after completion of PD training. Patients also return to the PD training centre one week later for a review of their technique and to review how they are coping.

Schedule

Training is done on consecutive days, with care to limit interruptions to no more than two days. One study suggested that training sessions of one to two hours per day reduce peritonitis rates [3], although practices in the world vary [1]. In our hospital, learners typically have three hours of training in the morning followed by a lunch break of one to two hours and another session of three hours in the afternoon. Short breaks are given throughout each session, and whenever requested.

Formal PD training is held 14 days after catheter implantation although exceptions are made for patients undergoing urgent start PD. One study showed that the highest peritonitis rates are associated with training within the first 10 days of catheter insertion [2].

The trainer sets the objectives for each day and lays out the knowledge and procedural skills acquisition goal each day. Unlike the ISPD training syllabus [1], autonomy is given to the trainer to modify the objectives for each day to suit the learner’s needs. For example, more topics can be covered in each session if the learner is fast and able to provide satisfactory feedback that there is sufficient knowledge and skills retention. As such, our hospital’s training log in Appendix 2 does not break down the topics by day but allows the trainer to record which topics are covered on each day. This allows trainers and learners to adapt to each individual needs and focus their efforts on certain segments, should they need it.

In our hospital, trainers adopt a “learning by doing” approach as part of their teaching pedagogy. Trainers will first teach procedures and concepts, alternating with discussions and questions. Learners are then given the opportunity to practice, rehearse, and role-play if appropriate. The topics covered also gradually progress from simple tasks (such as ensuring good personal and hand hygiene) to more complex ones (like management of infectious complications).

Learners and patients also have access to a PD walk-in clinic on weekdays during working hours and a 24-hour hotline to seek advice. Retraining is not routinely scheduled. However, it can be arranged should the need arise. 

Data collection

Clinical information, including patient demographics, PD training details, infection rates, and dropout rates, was extracted from the hospital's electronic health records using a standardized data mapping protocol. The data collection period covered all relevant medical records from the initiation of the PD program at our hospital from September 2018 to July 2023.

Variables

The primary outcomes measured were PD dropout rates and PD-related infections which encompasses PD peritonitis and PD exit site infection. Exit site infections and PD peritonitis episodes were diagnosed based on ISPD guidelines.

Efforts to address bias

We implemented robust measures to validate the accuracy of the extracted data and mitigate potential biases. Two independent investigators systematically cross-referenced the data against the original source documentation to verify its precision and minimize the introduction of bias. Additionally, we adhered to standardized data collection protocols and conducted rigorous data validation procedures to address potential sources of bias, such as selection bias and information bias.

Sample size determination

The sample size for this study (n = 99 patients) was determined based on the total number of end-stage kidney disease patients who met the inclusion criteria during the recruitment timeframe. Given that our hospital is a regional center with a steadily growing PD program, we included all incident adult patients aged 18 years and above who initiated peritoneal dialysis at our hospital from September 2018 to July 2023. This approach ensured a comprehensive analysis of our PD training program's impact on a representative patient population without the need for a pre-determined sample size calculation.

Statistical analysis

Descriptive statistics are presented as counts (n) and percentages (%), or as mean ± standard deviation (SD). Continuous variables were assessed using Student's t-test for parametric data. Fisher's exact test was applied for categorical events with fewer than five observations, while the chi-squared test was used for other categorical variables. Statistical significance was set at p < 0.05. Binary logistic regression modeling was performed to identify potential independent predictors influencing the dropout rate. All statistical analyses were performed using IBM SPSS Statistics, version 27.0 (IBM Corp., Armonk, NY, USA).

This study was approved by the Institutional Review Board (IRB).

Handling missing data

The study did not have any missing data for the variables analysed, as all relevant information was obtained from the electronic health records.

## Results

**Figure 1:**
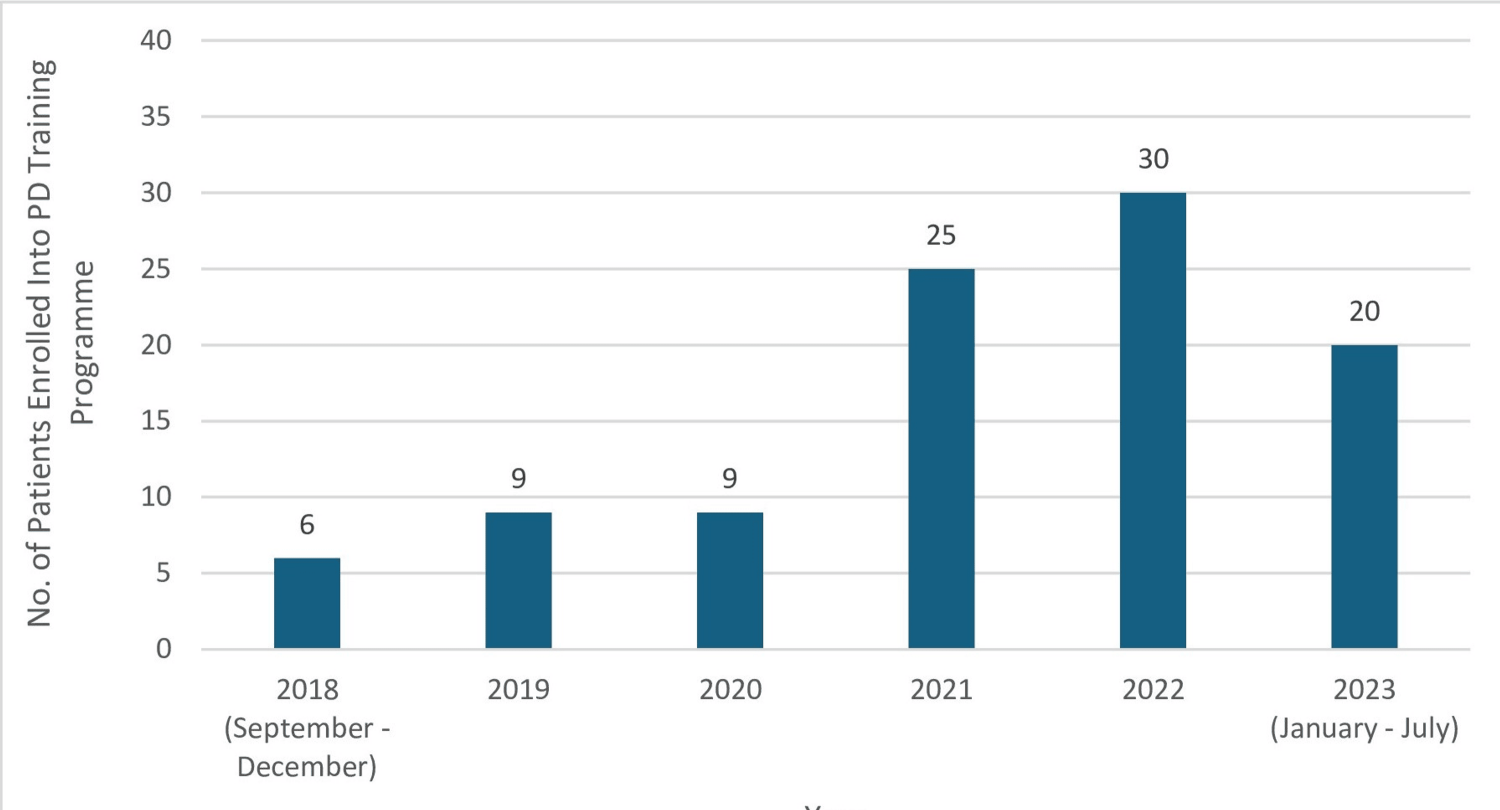
Number of patients enrolled into peritoneal dialysis training programme per year in our hospital PD= Peritoneal Dialysis

Our peritoneal dialysis unit, established in September 2018, has progressively expanded to accommodate 121 patients as of July 2023. The annual number of patients newly initiating PD has shown a consistent increase, with the exception of 2020, when elective services were suspended due to the COVID-19 pandemic. Among these 121 patients, 99 were incident PD patients who had their PD catheters inserted and completed their PD training program at our hospital. This excludes 20 patients who transferred from other institutions and 2 patients who discontinued PD before completing three months of therapy. Figure 1 illustrates the annual enrollment of PD patients in our Peritoneal Dialysis Training Programme. This retrospective cohort study concentrated on these 99 ESKD patients who completed the entire PD training program at our institution.

The demographic and clinical characteristics of the included patients are shown in Table 1. The mean age was 58.63 years, with 55.6% being male. The primary causes of ESKD were diabetic nephropathy (56.6%), glomerulonephritis (24.2%), and hypertension (14.1%). Approximately 59.6% of the patients had diabetes mellitus, while other common comorbidities included hypertension (92.9%), ischemic heart disease (41.4%), and cerebrovascular accident (22.2%). The mean baseline estimated glomerular filtration rate (eGFR) was 7.05 mL/min/1.73m², and the mean albumin level was 35.92 g/L.

**Table 1:** Baseline Characteristics of The Study Cohort SD= standard deviation; eGFR= estimated glomerular filtration rate Continuous variables were assessed using Student's t-test for parametric data and data presented as Mean ± standard deviation (SD) Fisher's exact test was applied for categorical events with fewer than five observations, while the chi-squared test was used for other categorical variables. Data are presented as counts (n) and percentages (%).

Baseline characteristic	Patients Remained in the Programme, N (%)	Patients Dropped Out of the Programme, N (%)	p-value	Total
Numbers of patients	73 (74%)	26 (26%)		99
Body mass index (kg/m²), Mean ± SD	25.41 ± 5.62	25.83 ± 4.81	0.733	25.52 ± 5.40
Baseline eGFR, (mL/min/1.73m²), Mean ± SD	6.82 ± 3.16	7.69 ± 3.72	0.254	7.05 ± 3.32
Hemoglobin (g/dL), Mean ± SD	9.77 ± 1.52	9.95 ± 1.42	0.594	9.82 ± 1.50
Albumin (g/L), Mean ± SD	36.60 ± 5.18	34.00 ± 6.16	0.039	35.92 ± 5.54
Age (years), Mean ± SD	57.30 ± 16.64	62.35 ± 16.37	0.186	58.63 ± 16.64
Diabetes Mellitus	42 (57.5%)	17 (65.4%)	0.484	59 (59.6%)
Hypertension	53 (72.6%)	19 (73.1%)	0.101	92 (92.9%)
Cancer	1 (1.4%)	1 (3.8%)	0.441	2 (2.0%)
Cerebrovascular accident	17 (23.3%)	5 (19.2%)	0.669	22 (22.2%)
Dementia	2 (2.7%)	1 (3.8%)	0.777	3 (3.0%)
Etiology of end-stage kidney disease			0.074	
Diabetes mellitus	39 (53.4%)	17 (65.4%)		56 (56.6%)
Hypertension	6 (8.2%)	8 (30.8%)		14 (14.1%)
Glomerulonephritis	23 (31.5%)	1 (3.8%)		24 (24.2%)
Polycystic kidney disease	2 (2.7%)	0 (0.0%)		2 (2.0%)
Others	3 (4.1%)	0 (0.0%)		3 (3.0%)
Ischemic heart disease	28 (38.4%)	13 (50%)	0.301	41 (41.4%)
Male	37 (50.7%)	18 (69.2%)	0.102	55 (55.6%)
Race			0.087	
Chinese	56 (76.7%)	16 (61.5%)		72 (72.7%)
Indian	4 (5.5%)	0 (0.0%)		4 (4.0%)
Malays	12 (16.4%)	10 (38.5%)		22 (22.2%)
Others	1 (1.4%)	0 (0.0%)		1 (1.0%)

In the multivariate analysis, after adjusting for age, gender, comorbidities, body mass index, haemoglobin, and baseline eGFR, hypoalbuminemia was associated with an increased risk of dropout, with an OR= 0.951, 95% CI: 0.875-1.033, but this association was not statistically significant.

Dropout rates over the study period are detailed in Table 2. In 2019, the dropout rate was 40%, with three out of six cases being PD-related. This rate improved to 17% in 2020, with only one PD-related dropout. By 2021, the dropout rate had further decreased to 10%, with no PD-related dropouts, and it remained steady at 10% in 2022, again with no PD-related dropouts. In the first half of 2023, the dropout rate dropped to 7%, with two out of six cases being PD-related. In this context, PD-related issues refer to PD peritonitis or exit site infections, while non-PD-related factors include events such as death, opted conservative care, or renal transplantation. This improvement was statistically significant, with an OR = 0.45, 95% CI = 0.49 to 0.84, p = 0.010. These improvements likely reflect enhanced patient education, better support systems, and more effective management of comorbid conditions.

**Table 2:** Peritoneal Dialysis Drop-Out Rates PD= Peritoneal Dialysis; HD = Haemodialysis PD related were due to PD peritonitis or PD exit site infection Non-PD related due to patient underwent renal transplantation, opted conservative care or death

Year	Patient Newly Enrolled into PD programme (No.)	Drop Out (No.)	Conversion to HD (No.)	Death (No.)	Transplantation (No.)	Conservative (No.)	Drop Out Rate (Drop Out/ Incident PD Patients %)
2018 (September- December)	6	0	0	0	0		0
2019	9	6	4 (3 PD related)	2	0		40% (6/15)
2020	9	3	1 (PD related)	2	0		17% (3/18)
2021	25	4	1 (Non-PD related)	2	0	1	10% (4/40)
2022	30	7	1 (Non-PD related)	4	2		10% (7/66)
2023 (January- July)	20	6	3 (2 PD related)	2	1		7% (6/79)

**Figure 2:**
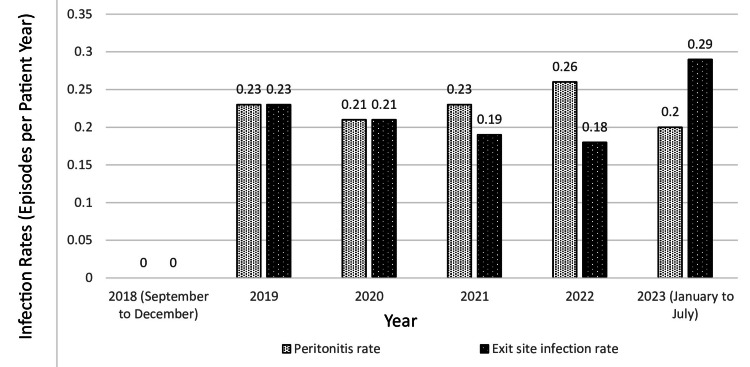
Peritoneal Dialysis Peritonitis and Exit Site Infection Rates Calculation for peritonitis rate 1) Total number CAPD/APD patient days at risk / 365 days per year = years experience 2) Number of episodes of peritonitis/number of years experience = episodes per patient year Calculation for exit site infection rate 1) Total number CAPD/APD patient days at risk / 365 days per year = years experience 2)  Number of episodes of exit site infection per year /years experience CAPD= Continuous Ambulatory Peritoneal Dialysis; APD: Automated Peritoneal Dialysis

Peritonitis and exit site infection rates are shown in Figure 2. This illustrates the PD-related infection rates over the years in our hospital’s PD program. The peritonitis rates ranged from 0.2 to 0.26 episodes per patient-year, while exit site infection rates fluctuated between 0.18 and 0.29 episodes per year, consistent with international guidelines [8,10]. A notable increase in exit site infections from January to July 2023 may have been influenced by surgical factors associated with a less experienced surgeon.

## Discussion

The ISPD has established guidelines to ensure high standards of care in PD. Specifically, the ISPD recommends that overall exit site infection rates should not exceed 0.40 episodes per year at risk, and peritonitis rates should be less than 0.4 episodes per patient-year at risk [3,11,12]. These metrics are crucial for assessing the quality of PD care, as they directly impact patient outcomes and treatment sustainability. High peritonitis and dropout rates often indicate inadequate training, support, or clinical practices, necessitating continuous monitoring and improvement [8,9,13].

At our hospital, our PD program has consistently adhered to these recommended infection rates. Our peritonitis rates have ranged from 0.2 to 0.26 episodes per patient year, while our exit site infection rates have fluctuated between 0.18 and 0.29 episodes per year [8,9]. A notable increase in exit site infections from January to July 2023 may have been influenced by surgical factors associated with a less experienced surgeon., highlighting the importance of surgical expertise in infection control [3,8]. These infection rates are illustrated in Graph 2, providing a comprehensive overview of PD-related infection trends over the years within our program. Our study results were shared in our local Renal Outlook from the National Kidney Foundation in Singapore to provide insights into our PD training program for the kidney care community within Singapore [14].

In addition to infection rates, dropout rates are another critical indicator of the quality of a PD program. Since the inception of our program in September 2018, we have observed a significant improvement in dropout rates, largely attributed to non-PD-related causes [8,13]. In 2019, the dropout rate was alarmingly high at 40%, with half of the dropouts being PD-related. However, through enhanced patient education, robust support systems, and effective management of comorbid conditions, we reduced the dropout rate to 17% in 2020, with only one PD-related dropout. This positive trend continued, with the dropout rate further decreasing to 10% in both 2021 and 2022, with no PD-related dropouts. The first half of 2023 saw the dropout rate drop even further to 7%, although two out of six cases were PD-related. These statistics are detailed in Table 2, reflecting the efficacy of our interventions and continuous quality improvement efforts.

The improvements in our dropout rates underscore the importance of comprehensive patient education and support [15,16]. Our training program emphasizes the correct technique for PD catheter care, recognizing signs of infection early, and managing common complications. Additionally, we have implemented regular follow-up visits and established a hotline for patients to seek immediate assistance, thereby reducing the likelihood of complications leading to dropout [17,18]. These measures have proven effective in maintaining patient engagement and adherence to the PD regimen.

While our tailored training program has successfully reduced dropout and infection rates, it’s worth noting that the one-to-one training recommended by ISPD is labor-intensive and can sometimes exceed the suggested hours [1]. Moving forward, gathering feedback from both patients and nurses could be beneficial, considering the significant man-hours invested by both parties.

Overall, our experience highlights the importance of targeted training programs and support systems in managing PD effectively. By maintaining low infection rates and reducing dropout rates, we have demonstrated that with the right resources and strategies, it is possible to achieve high standards of care in PD. Our findings contribute to the broader discourse on PD management, offering valuable insights into best practices that can be adopted by other institutions aiming to improve their PD programs.

Limitations

This study has several limitations, including its retrospective design, which may introduce information bias despite our efforts to validate data accuracy. Additionally, the single-center setting and relatively small sample size may limit the generalisability of our findings to other populations and healthcare systems. Finally, while adjustments were made for several potential confounders, the exclusion of certain patients and the possibility of unmeasured variables could still influence the results

Generalisability

The results of this study are based on data from a single regional hospital in Singapore, which may limit the generalisability to other settings. However, the findings are relevant to similar healthcare environments where structured PD training programs are implemented. The consistent adherence to ISPD guidelines and the observed improvements in patient outcomes suggest that our tailored training program can be adapted and applied in other institutions to achieve comparable results. Future research involving multiple centers would further validate the generalisability of these findings.

## Conclusions

Our modified PD training curriculum has proven to be a safe and effective method for training patients and caregivers in peritoneal dialysis (PD) delivery, as evidenced by our adherence to audit standards for complication incidences. The incidences of complications, including PD peritonitis and exit site infections, met the guidelines, indicating the robustness of our training program.

Moving forward, our hospital will continue to monitor outcomes such as infectious complications and dropout rates. Further studies are recommended to evaluate both trainer and learner satisfaction to identify potential areas for enhancement. Other parameters worth monitoring include formal assessments of barriers to learning PD and knowledge retention rates over time. Additionally, we will continue to monitor peritonitis rates to assess the long-term impact of our training pedagogy.

By maintaining rigorous oversight and continuously refining our training methods, we aim to sustain and further improve the quality of care provided to PD patients. These efforts will ensure that our program remains responsive to patient needs and aligned with best practices in PD management, contributing to continuous quality improvement.

## Appendices

Appendix 1

**Table 3:** Section of Peritoneal Dialysis Training Log for Patients PD= Peritoneal Dialysis

Theoretical Knowledge						
Date of Training:						Remarks
Main Topic	Specific Topics	Day 1	Day 2	Day 3	Day 4	
Functions of the kidneys	Understand how the kidneys work					
	Understand what happens when the kidney fail					
Introduction to PD	Understand how PD works					
	Understand how the peritoneum act as a filter					
	Understand what is osmosis and ultrafiltration					
	Understand the concept of drain, fill and dwell.					

Appendix 2

**Table 4:** Peritoneal dialysis teaching syllabus summary PD=Peritoneal Dialysis

Day	Duration	Topics Taught
Introductory Phase		
Day 0: (Inpatient)	1 hour	Introduction to PD
Post PD Catheter Insertion Day 1		Dressing care
Discharge Day		PD Catheter and exit site care
		Daily life modifications + Integrating home life with PD: Eg. Avoidance of constipation Fluid management,
		PD Team’s support information: Including on call and walk-in clinic services
Day 5 and Day 10: (Outpatient)	1 hour	Patient returns to PD training center for flushing of PD catheter and exit site care on Post-Op Day 5
		Personal/hand hygiene and aseptic technique,
		Kidney and PD physiology,
		Assessment of topics taught previously: Eg. Exit site care
Formal Training Phase		
Day 15: (Outpatient)	6 hours	Course overview
Day 1 of formal PD training		Revision and assessment of knowledge of previous topics: Eg. Hand hygiene PD catheter and exit site care
Day 16: (Outpatient)	6 hours	PD exchange: Patients who choose automated peritoneal dialysis (APD) will be taught to do PD with both the cycler and “manual”
Day 2 of formal PD training		Documentation of vital signs and PD record book
Day 17: (Outpatient)	6 hours (Inclusive of 2 hours of dietician, pharmacist and vendor review)	Inflow and outflow issues
Day 3 of formal PD training		Contamination management
		Infectious complications including PD peritonitis exit site and tunneled tract infection
		Fluid management
		Patient is also reviewed by a nephrologist to reconcile therapy plan and establish PD home regime,
		Training also includes: (Typically about 2h)
		Information on financial claims/ aid and logistical support information by vendor
		Dietary review by dietician
		Medication compliance and pharmacist review
Day 18: (Outpatient)	3 hours	Review of topics
Day 4 of formal PD training		Assessment of learner’s ability to self-care/ be a caregiver independently
